# Iron deficiency in plants: an insight from proteomic approaches

**DOI:** 10.3389/fpls.2013.00254

**Published:** 2013-07-25

**Authors:** Ana-Flor López-Millán, Michael A. Grusak, Anunciación Abadía, Javier Abadía

**Affiliations:** ^1^Plant Nutrition Department, Aula Dei Experimental Station (CSIC)Zaragoza, Spain; ^2^Department of Pediatrics, USDA-ARS Children's Nutrition Research Center, Baylor College of MedicineHouston, TX, USA

**Keywords:** iron, metabolism, proteomics, root, thylakoid

## Abstract

Iron (Fe) deficiency chlorosis is a major nutritional disorder for crops growing in calcareous soils, and causes decreases in vegetative growth as well as marked yield and quality losses. With the advances in mass spectrometry techniques, a substantial body of knowledge has arisen on the changes in the protein profiles of different plant parts and compartments as a result of Fe deficiency. Changes in the protein profile of thylakoids from several species have been investigated using gel-based two-dimensional electrophoresis approaches, and the same techniques have been used to investigate changes in the root proteome profiles of tomato (*Solanum lycopersicum*), sugar beet (*Beta vulgaris*), cucumber (*Cucumis sativus*), *Medicago truncatula* and a *Prunus* rootstock. High throughput proteomic studies have also been published using Fe-deficient *Arabidopsis thaliana* roots and thylakoids. This review summarizes the major conclusions derived from these “-omic” approaches with respect to metabolic changes occurring with Fe deficiency, and highlights future research directions in this field. A better understanding of the mechanisms involved in root Fe homeostasis from a holistic point of view may strengthen our ability to enhance Fe-deficiency tolerance responses in plants of agronomic interest.

## Introduction

Iron (Fe) is the sixth most abundant element in the universe and the fourth most abundant in the earth's crust (Morgan and Anders, 1980). For most living organisms on earth, Fe is an essential element. However, its chemical properties in oxygenated environments limit its availability. As a consequence, Fe deficiency is a widespread phenomenon among the animal and plant kingdoms. According to the WHO, anemia (including Fe-deficiency induced anemia) affects more than 2 billion people worldwide (McLean et al., 2009). In crops, and especially in those grown on calcareous soils, Fe deficiency is a major nutritional disorder that causes decreases in vegetative growth and marked yield and quality losses (Abadía et al., 2011). A substantial body of knowledge has arisen during the genomic era with regard to the molecular aspects of plant Fe acquisition mechanisms that are induced in response to limited Fe availability (Palmer and Guerinot, 2009; Schmidt and Buckhout, 2011; Hindt and Guerinot, 2012). In all plants except grasses, the adaptation mechanism to Fe limitation suggests the need for increased reducing power (NADH) for the root plasma membrane (PM) Fe reductase, and increased energy (ATP) for the PM H^+^-ATPase (Römheld and Marschner, 1983). Proteomic approaches are an excellent tool to elucidate the general metabolic reprogramming needed to sustain these requirements. Understanding how plants respond to Fe limitation at the proteomic level provides a new layer of information on Fe homeostasis processes that can ultimately help to reduce the effects of Fe deficiency in crops. This review summarizes, in a comprehensive manner, the major conclusions regarding metabolic adaptations that occur under Fe deficiency conditions in roots and thylakoids of several plant species, and highlights future research directions in the field. Also, the review provides a clear overview on the limitations of the different proteomic techniques available to study a prevalent abiotic stress case in crops.

## Changes induced by fe deficiency in the root proteome

When Fe is scarce, Strategy I plants develop morphological and biochemical changes leading to an increase in their Fe acquisition capacity. Morphological changes include swelling of root tips and formation of lateral roots, root hairs, and transfer cells that increase the root's surface area. Biochemical changes result in an increased ability to acquire Fe, and include the induction of a plasma-membrane Fe(III)-reductase and an Fe(II) transporter, an enhanced proton extrusion capacity, and the release of low molecular weight compounds such as carboxylates, flavins and phenolic compounds (Abadía et al., 2011).

The most common approach used to study changes in the root proteome upon Fe starvation has been a gel-based approach, using two-dimensional (2-DE) IEF SDS/PAGE electrophoresis. All of the studies reviewed here have been conducted using root extracts of Strategy I plants, whereas proteomic studies in Strategy II plants have only been conducted in root plasma membrane of maize (Hopff et al., 2013). Small gels (7 cm) were used in studies involving *Beta vulgaris* (Rellán-Álvarez et al., 2010), *Medicago truncatula* (Rodríguez-Celma et al., 2011a) and *Prunus dulcis* × *Prunus persica* (Rodríguez-Celma et al., 2013a), whereas larger gels were used for *Cucumis sativus* (24 cm, Donnini et al., 2010) and *Solanum lycopersicum* (24 cm in Li et al., 2008 and 13 cm in Brumbarova et al., 2008). When using small gels the average number of proteins detected in root extracts ranged between 140 for *B. vulgaris* to ~335 for *P. dulcis* × *P. persica* and *M. truncatula*, whereas in larger gels the number of spots ranged from 1400 in *S. lycopersicum* to 2200 in *C. sativus*. Independently of the total number of spots, the number of spots whose relative abundance changed significantly as a result of the different Fe deficiency treatments was within the same range among plant species, with 17, 31, and 61 spots for *P. dulcis* × *P. persica, M. truncatula* and *B. vulgaris*, 57 for *C. sativus*, and 41 (Li et al., 2008) and 24 (Brumbarova et al., 2008) for *S. lycopersicum*, respectively. The protein identification rates ranged between 71 and 95%, except for *B. vulgaris* where only 36% of the proteins changing in abundance upon Fe limitation were identified, underlying the importance of using plant species with sufficient genomic information for proteomic studies. In *A. thaliana* root extracts, a high-throughput study of changes induced by Fe limitation was performed using HPLC-MS and iTRAQ (Isobaric Tag for Relative and Absolute Quantification). In this study, 4454 proteins were identified in *A. thaliana* root extracts and 2882 were reliably quantified; from these, a subset of 101 proteins were identified whose abundance changed upon Fe deficiency (Lan et al., 2011). Finally, a phosphoproteomics study with *A. thaliana* found that among 425 phosphorylated proteins, 45 changed in abundance with Fe deficiency (Lan et al., 2012).

The comparison of proteomes from multiple plant species is not straightforward. This is because of difficulties in handling homology between species, in particular when public genomic data sets are not comprehensive, or in some cases are lacking, as it occurs with *B. vulgaris*. Furthermore, the reliability of protein identification using database comparisons always has some uncertainty and clearly depends on the stringency of the criteria selected for positive identification, including number of peptides matched and sequence coverage. In addition, because any given proteomic profile is a snapshot of a dynamic process, differences in plant developmental stage and in the manner Fe limitation is imposed can likely lead to final protein profile pictures that may differ significantly. Also, we should keep in mind that changes in spot intensity can be related to post-translational modifications, adding a new layer of complexity to the interpretation of proteomic studies. As a compromise, we used an approach where identified proteins found to change in each plant species were matched to proteins existing in *A. thaliana*. BLAST searches were run for every protein species found in the different studies and those matches with *E*-values below e^−30^ were retained as positive *Arabidopsis* orthologs; if any of the accession numbers overlapped between two or more entries of the same plant species, they were considered redundant and only a single representative entry was retained (Table S1). Also, in an attempt to reduce variability associated with differences in the treatments, the proteomes of *B. vulgaris* and *M. truncatula* plants grown in the absence of Fe, but in the presence of CaCO_3_, were in principle excluded from the comparison (although data are also provided in Table S1).

**Figure 1:**
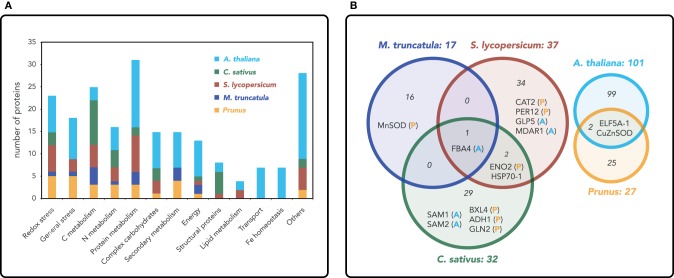
**(A)** Functional classification of proteins in root extracts that shows changes in relative abundance as a result of Fe deficiency. The number of proteins and functional assignments were obtained from proteomic studies in roots of *Arabidopsis thaliana* (yellow; Lan et al., 2011), *Cucumis sativus* (green; Donnini et al., 2010), *Solanum lycopersicum* (red; Brumbarova et al., 2008; Li et al., 2008), *Medicago truncatula* (blue; Rodríguez-Celma et al., 2011a) and the Prunus rootstock *Prunus dulcis* × *Prunus persica* (orange; Rodríguez-Celma et al., 2013a). **(B)** Proteins with changes in relative abundance as a result of Fe deficiency in two or more plant species are shown in a Venn diagram. Since the comparison of five species in a single Venn diagram was not fully clear, we used two Venn graphs: in the one in the left, a comparison is made between *C. sativus, S. lycopersicum*, and *M. truncatula* (letters next to the protein species acronyms indicate whether changes also occur in *A. thaliana* and the Prunus rootstock), whereas the one in the right shows the contrast between *A. thaliana* and the Prunus rootstock. Numbers within the circles indicate the total number of proteins changing in relative abundance as a result of Fe deficiency in each plant species.

Based on this approach, 199 non-redundant *Arabidopsis* orthologs were found to change in relative abundance with Fe deficiency: 101, 17, 37, 27, and 32 of them were derived from the *Arabidopsis* (Lan et al., 2011), *M. truncatula, S. lycopersicum, P. dulcis* × *P. persica*, and *C*. *sativus* studies, respectively. These non-redundant root proteins and the results from the BLAST searches are listed in Table S1. Results showing proteins grouped by function (generally as described by authors, although with some modifications) are summarized in Figure 1A and Table S2 (in the latter case with red and green backgrounds indicating proteins increasing or decreasing in relative abundance, respectively). This comparison did not yield a single specific protein species changing in all plant species as a result of Fe limitation, whereas only 15 proteins did show changes in two or more plant species (Figure 1B; Table S2). Among them, six were related to stress defense, three were related to C metabolism, and three to N metabolism. The remaining three protein species were a β-xylosidase involved in cell wall modification, the translation initiation factor ELF5A-1, and the chaperone HSP70-1. In spite of the low number of common protein overlaps, changes in metabolic pathways do follow a similar trend. It should be noted that changes in Fe homeostasis and transport proteins upon Fe deficiency were only detected in *Arabidopsis* using HPLC-MS, because most of these proteins are membrane associated and therefore difficult to solubilize and detect with gel-based 2-DE approaches.

### Common changes among plant species

#### Oxidative stress and defense

Oxidative stress was the category containing more specific proteins (five) changing as a result of Fe deficiency in more than one plant species. These changes included increases in superoxide dismutases (CuZnSOD and MnSOD), monodehydroascorbate reductase (MDAR1), peroxidase 12 (PER12) and a decrease in catalase (CAT-2) (Figure 1B; Table S2). These observations point to the strong impact of Fe deficiency on redox homeostasis, not only because free Fe ions induce reactive oxygen species (ROS) formation via Fenton reactions, but also because many proteins involved in oxidative stress such as peroxidases and catalase are Fe-containing proteins. Some of the changes observed, such as the compensatory mechanism of the SOD isoenzymes in *P. dulcis* × *P. persica, M. truncatula* and *Arabidopsis*, as well as the decrease in catalase in *S. lycopersicum* and *P. dulcis* × *P. persica*, are likely associated with the decrease in Fe availability. The increases and decreases reported in some of the plant species for the different peroxidase species (i.e., increases in PER12, PER3 and decreases in PER53 and PER21, all of them containing Fe), indicate the complex interplay between Fe and redox homeostasis mechanisms. Increases in MDAR (in *S. lycopersicum* and *Arabidopsis*) and ascorbate peroxidase (APX; in *S. lycopersicum*) also indicate an induction of the glutathione-ascorbate cycle in response to Fe deficiency.

With regard to defense proteins, only GLP5 (Germin-Like-Protein 5) increased in two species, *S. lycopersicum* and *Arabidopsis* (Figure 1B; Table S2). This protein is plasmodesmata-localized and it is involved in regulating primary root growth by controlling phloem-mediated allocation of resources between the primary and lateral root meristems (Ham et al., 2012). Six glutathione S transferase (GST) proteins changed as a result of Fe deficiency, although no specific overlaps were observed between plant species. The GSTs decreasing in abundance in *P. dulcis* × *P. persica* (GSTF9 and GSTF4) belong to the phi class, whereas the GSTs increasing in abundance belong to the lambda (GSTL1 in *Arabidopsis* and GSTL3 in *P. dulcis* × *P. persica*) and tau (GSTU19 in *M. truncatula* and GSTU25 in *Arabidopsis*) classes. The phi class has been linked to tolerance to oxidative stress, whereas the lambda family likely has an oxidoreductase activity and the tau subclass of GST binds fatty acid derivatives (Dixon and Edwards, 2010). Even though the specific functions of these GSTs are not well known, these proteomic data suggest that glutathionylation could be a common detoxifying strategy in Fe-deficient roots.

#### C metabolism

Carbon metabolism, and in particular glycolysis, is the metabolic pathway showing the most consistent changes with Fe deficiency at the proteomic level within all plant species studied (also including those grown in the presence of CaCO_3_) (Figure 1B, Table S2). Two or more proteins belonging to this pathway increased in abundance in all plant species, except for *Arabidopsis*, where only one protein increased, a fructose bisphosphate aldolase for which increases were also found in *S. lycopersicum* and *C*. *sativus* (and also in *M*. *truncatula* and *B*. *vulgaris* grown with CaCO_3_). Enolase also showed a consistent increase in all species (in *M. truncatula* in the presence of CaCO_3_) except *Arabidopsis*. Furthermore, these two proteins were the only ones increasing with Fe deficiency in three of the five plant species studied, highlighting the fact that the metabolic reprogramming of carbon metabolism that occurs upon Fe deficiency is well conserved among species. Increases in the abundance of enzymes from the TCA cycle with Fe deficiency were found in *C. sativus* and *M*. *truncatula*, whereas an increase in PEPC was detected in *S*. *lycopersicum*. These proteomic results are in agreement with transcriptomic and biochemical studies that report increases in glycolysis and TCA cycle enzymes upon Fe limitation (see Zocchi, 2006 for a review). Increases in the activity of these pathways could (1) fulfill the increased demand for reducing power and ATP from the up-regulated Fe acquisition mechanisms, including the PM Fe reductase and ATPase, and (2) account for the increases in carboxylates consistently reported in Fe-deficient roots. These holistic proteomic approaches highlight the importance of general metabolic pathways in the elicitation of the stress response mechanisms. In only one case, a protein of the TCA cycle, aconitase in *C. sativus*, showed a relative decrease in abundance with Fe deficiency. Although aconitase is an Fe containing protein, this decrease has only been reported for *C. sativus*, whereas contrasting changes in aconitase activity as a result of Fe deficiency have been reported in other plant species (Abadía et al., 2002). These apparent inconsistencies with regard to aconitase could be explained by the different ways Fe treatments were imposed: in the *C. sativus* study, seedlings were always grown in the absence of Fe, whereas in the other studies plants were pre-grown with Fe before imposing the Fe-deficiency treatment.

Two enzymes involved in fermentation, alcohol dehydrogenase in *P. dulcis* × *P. persica* and *C. sativus* and formate dehydrogenase in *S. lycopersicum* (and in *B. vulgaris* grown in the presence of CaCO_3_) increased in relative abundance in Fe-deficient roots. These results are also in agreement with previous studies, and have been associated with low intracellular O_2_ caused by an increased consumption of O_2_ in Fe-deficient roots (López-Millán et al., 2000, 2009).

#### N metabolism

Iron deficiency also has an impact on N metabolism. Three proteins related to N metabolism changed in at least two plant species: glutamine synthetase (GLN2) increased in *P. dulcis* × *P. persica* and *C. sativus*, S-adenosylmethionine synthase 1 (SAM 1) increased in *C. sativus* and *Arabidopsis*, and SAM2 decreased in *C. sativus* and increased in *Arabidopsis* (Figure 1B, Table S2). In some species, increases were also observed in enzymes involved in ammonia release [urease (UREG) and dihydrolipoamide dehydrogenase (LPD1) in *M. truncatula* and an omega amidase in *C. sativus*], assimilation (GLN1 in *Arabidopsis*, GLN2 in *P. dulcis* × *P. persica* and *C. sativus*) and amino group transfer (alanine aminotransferase in *C. sativus* and aspartate aminotransferase in *M. truncatula* grown with CaCO_3_). Overall, these changes indicate an increase in N recycling that could help to overcome the reduced N assimilation caused by a decrease in the Fe-containing enzyme nitrite reductase. Decreases in nitrite reductase have been found in *M. truncatula* and *S. lycopersicum* at the protein level and in *C. sativus* at the biochemical level (Borlotti et al., 2012).

Fe deficiency has a conserved impact on SAM metabolism among plant species. Increases in proteins involved in SAM synthesis have been found at the proteomic level in *S. lycopersicum, M. truncatula, C. sativus* and *Arabidopsis*. Several authors have already pointed out the central role of SAM in the Fe-deficiency response, not only as a methyl group donor but also as a precursor for the synthesis of nicotianamine, an important Fe chelator, and ethylene, a hormone with a role in the regulation of Fe acquisition in roots (Waters et al., 2007; Donnini et al., 2010; Lan et al., 2011).

### Species- or treatment-specific changes

#### Cell wall

A significant number of proteins involved in the metabolism of glycosyl compounds are Fe-responsive in all plant species except in *M. truncatula* (one in *P. dulcis* × *P. persica*, four in *S. lycopersicum*, three in *C. sativus* and eight in *Arabidopsis*), and most of them are localized in the apoplast, suggesting that Fe shortage leads to modifications in cell wall structure and metabolism. Although a more detailed biochemical study is needed to understand these changes, these responses to Fe deficiency seem to be species-specific. In *C. sativus*, decreases were measured for two enzymes involved in the hydrolysis of O-glycosyl compounds (1,4-β-xylosidase and UDP-glucose dehydrogenase) and an invertase, whereas in *P. dulcis* × *P. persica* only 1,4-β-xylosidase was found to decrease. In *Arabidopsis*, in addition to decreases in proteins involved in the transfer of glycosyl groups, lignin catabolism, and hydrolysis, increases were measured in five more proteins: four involved in lignin or cellulose biosynthesis and a β-glucosidase involved in cellulose degradation. A similar situation was observed in *S. lycopersicum*, with two cell wall-related proteins increasing and two decreasing. In the studies with *C. sativus*, the authors explained these changes as a decrease in cell wall biosynthesis that could potentially lead to increased carbon flux toward other metabolic processes such as glycolysis (Donnini et al., 2010); however, the nature of the proteins identified may also reflect hydrolysis of the cell wall as a means to supply carbon skeletons. Irrespective of the specific changes occurring, it can be concluded that cell wall remodeling occurs upon Fe limitation; whereas this may provide carbon for glycolysis or other processes, it is also likely to cause changes in the dynamics of intercellular metabolite trafficking.

#### Secondary metabolism

An interesting observation highlighted by our comparison relates to the species-specific changes observed in proteins of secondary metabolism. Even though no overlaps were seen among proteins changing in abundance with Fe deficiency in the different plant species, proteins participating in the riboflavin synthesis pathway increased in *M. truncatula* (and in *B. vulgaris* grown in the presence of CaCO_3_), whereas increases in several proteins involved in the phenylpropanoid pathway were observed in *Arabidopsis* roots and in isoprenoid and flavonoid synthesis in *P. dulcis* × *P. persica*. These proteomic results are in agreement with biochemical and transcriptomic data (Rellán-Álvarez et al., 2010; Lan et al., 2011; Rodríguez-Celma et al., 2011b, 2013b) and suggest that Fe deficiency induces the synthesis of different secondary metabolites in roots whose function in the Fe deficiency response deserves further attention.

#### Energy and ATP-coupled transport processes

Contrasting results are reported in root proteomes regarding energy-related proteins. In *Arabidopsis*, significant increases in the abundance of proteins participating in the mitochondrial transport chain, including several subunits of complex I, were observed upon Fe deficiency, whereas in *M. truncatula* and *C. sativus*, decreases were found for a subunit of complex I and in subunit B of the mitochondrial ATPase. Also, the abundance of two vacuolar ATPases was decreased in Fe-deficient *Arabidopsis*, whereas a higher abundance was reported for a vacuolar ATPase in Fe-deficient *S. lycopersicum*. The decrease in complex I has been confirmed at the biochemical level also in *C. sativus* (Vigani et al., 2009; Vigani, 2012). These apparent discrepancies could be species-specific or may be explained by the different growing conditions, because *Arabidopsis* was grown in agar under continuous illumination conditions (including the root) that can alter electron transfer reactions.

#### Protein metabolism

It is more difficult to extract general conclusions about changes in protein metabolism related processes induced by Fe deficiency. In *Arabidopsis*, several translation elongation and initiation factors increased in abundance upon Fe limitation, and abundance decreases were also found for some ribosomal components (Lan et al., 2011). In *P. dulcis* × *P. persica*, an abundance increase in a translation initiation factor was found, but similar changes were not observed in other species. In two species, *M. truncatula* and *S. lycopersicum*, an increase in proteolysis may take place upon Fe-deficiency based on increases measured in some proteases and in the reorganization of the proteasome machinery. In *C. sativus*, no changes were measured in proteolytic enzymes, but decreases were observed in structural proteins such as actin and globulins. The authors hypothesized that these proteins could be used as a source of C and N during Fe-deficient conditions that cause macronutrient starvation (Donnini et al., 2010).

## The shoot proteome

Changes induced by Fe deficiency in the shoot proteome have only been reported for *S. lycopersicum* (Herbik et al., 1996). In that study, although apparent differences were observed in the protein profiles of Fe-sufficient and Fe-deficient leaves, protein identification was not pursued because the aim of the work was to identify differences between a mutant *S. lycopersicum* genotype (chloronerva) and wild type.

## The thylakoid proteome

Thylakoid membranes contain the multiprotein photosynthetic complexes photosystems I and II, which include the reaction centers responsible for converting light energy into chemical bond energy, as well as cytochrome b_6_/f and ATPase complexes. Fe-deficient plants have a reduced number of granal and stromal lamellae per chloroplast and decreased amounts of many thylakoid membrane components including proteins, electron carriers and lipids and this is consistent with marked decreases in photosynthetic rates (Terry, 1980; Terry and Abadía, 1986; Morales et al., 1990).

Changes induced in the thylakoid proteome by Fe-deficiency have been studied in *B. vulgaris* and *Spinacia oleracea* using 2-DE blue native (BN) SDS/PAGE in combination with mass spectrometry (Andaluz et al., 2006; Timperio et al., 2007) and by HPLC and tandem mass spectrometry in *A. thaliana* (Laganowsky et al., 2009). These studies clearly demonstrate that Fe deficiency causes decreases in the relative amounts of electron transfer proteins, including the core and light harvesting components of the PSI and PSII complexes and in cytochrome b6/f, with the largest decreases seen in PSI. The time course studies performed in *S. oleracea* also indicate that Fe deficiency affects supercomplex organization, by inducing a transient decrease in trimeric and dimeric organization of the light harvesting complex of PSII. These changes might constitute an adaptive strategy to facilitate energy dissipation in Fe-deficient plants (Timperio et al., 2007).

Contrasting results were obtained for ATP synthase, showing no Fe-deficiency induced changes in *B. vulgaris* and *A. thaliana* and decreases in *S. oleracea*. Also, the relative amounts of proteins involved in leaf C fixation, including Rubisco, increased in *B. vulgaris*, whereas Rubisco decreased significantly in *S. oleracea*. These results may be species-specific or more likely are related to plant culture conditions, because *S. oleracea* was grown without Fe from germination, whereas *B. vulgaris* and *Arabidopsis* had a preculture period in the presence of Fe. Other changes observed in proteins present in thylakoid preparations upon Fe limitation included increases in enzymes involved in ROS protection, such as ascorbate peroxidase in *A. thaliana* and SOD in *B. vulgaris*.

The analyses of exact masses of thylakoid membrane proteins in *Arabidopsis* also revealed that several proteins undergo post-translational modifications upon Fe deficiency; for example, the PSII oxygen evolving complex in Fe-deficient *Arabidopsis* thylakoids was found mostly in its doubly phosphorylated form, while in Fe-sufficient samples the non-phosphorylated form was predominant (Laganowsky et al., 2009).

Results provided in these papers underscore that 2-DE BN SDS/PAGE and HPLC-MS are the techniques more suitable for proteomic studies involving membranes, because they allow the resolution of highly hydrophobic integral membrane proteins. However, the use of other approaches such as 2-DE IEF SDS/PAGE also provides useful complementary information about other sets of proteins tightly linked to membranes.

## Conclusion and future perspectives

As proteomic and transcriptomic techniques have evolved toward high-throughput methodologies such as iTRAQ or the new Illumina technology, it has become more evident that a strict correlation does not exist between observed changes in protein and gene expression profiles. The comparison between changes in proteomic and transcriptomic profiles was out of the scope of this review; however, we have compared the datasets obtained from *Arabidopsis* Fe-deficient root extracts using iTRAQ and RNA sequencing. This comparison is only possible in *Arabidopsis*, where the genome is available and both studies have been reported; for other plant species, such as *Medicago*, the genome is still not finished and there are no reports of iTRAQ profiles. The total number of *Arabidopsis* transcripts identified was ~26 K, from which 2679 (10%) were differentially expressed upon Fe deficiency (Rodríguez-Celma et al., 2013b), whereas the total number of proteins identified by iTRAQ was 4454, from which 101 (2%) were differentially accumulated upon Fe deficiency (Lan et al., 2011). Among these, forty-seven protein species (50% of the differentially accumulated proteins) showed changes in both protein accumulation and gene expression (Table S3). The results of this comparison indicate that both omic approaches are complementary and that the gap in information between them is not only due to methodological limitations, but also reflects the complex regulation of stress responses such as Fe deficiency.

As shown in this review, proteomics has been a powerful tool in the elucidation of general metabolic rearrangements upon Fe deficiency. Different proteomic approaches have demonstrated that Fe deficiency has a profound impact on C metabolism and on the arrangement of the photosynthetic machinery, with many of these changes conserved amongst plant species. However, species- and treatment-specific changes have also been revealed that deserve further attention. The full potential of proteomic approaches is quite far from being fully exploited and detailed studies on specific changes unveiled with this holistic technique may yield new insights into the adaptation of plants to Fe deficiency. Future directions for proteomic studies should focus on the analysis of sub-proteomes crucial for Fe homeostasis, such as those of plant fluids, isolated organelles, and purified membrane preparations. Recent examples have been published for plasma membrane (Meisrimler et al., 2011; Hopff et al., 2013) and phloem sap (Lattanzio et al., 2013). These studies will help to elucidate the specific roles subcellular compartments may play in Fe homeostasis, and also will allow the discovery of proteins with new functions in these processes, which might remain hidden using other, less targeted approaches.

### Conflict of interest statement

The authors declare that the research was conducted in the absence of any commercial or financial relationships that could be construed as a potential conflict of interest.

## Supplementary material

Table S1 **Tables of BLAST results from proteins identified in roots from *Prunus dulcis* × *Prunus persica* (Rodríguez-Celma et al., 2013a), *Medicago truncatula* (Rodríguez-Celma et al., 2011a), *Beta vulgaris* (Rellán-Álvarez et al., 2010), *Cucumis sativus* (Donnini et al., 2010), and *Solanum lycopersicum* (Herbik et al., 1996; Brumbarova et al., 2008; Li et al., 2008)**. Spot nomenclature in the first column is that used in the original papers. BLAST searches were performed in the NCBI website (http://blast.ncbi.nlm.nih.gov/Blast.cgi) against the *Arabidopsis* database (taxid:3701) in March 2013. BLAST annotations were assigned as not hit when BLAST E-values were higher than 1e^−30^. If any of the accession numbers overlapped between two or more entries of the same plant species, they were considered redundant and only a single representative entry was retained.

Table S2 **Comparison of changes observed upon Fe deficiency in the non-redundant *Arabidopsis* root proteomes obtained from *Arabidopsis thaliana* (Lan et al., 2011) and from BLAST results in *Cucumis sativus, Solanum lycopersicum, Medicago truncatula* and *Prunus dulcis* × *Prunus persica***. Red and green backgrounds indicate increases and decreases, respectively, in protein abundance upon Fe deficiency. A blue background in the first column marks proteins showing changes in two or more plant species (not considering the *M. truncatula* and *B. vulgaris* treatments including CaCO_3_, which are included in the Table in the last two columns in white characters).

Table S3 **List of *Aradidopsis thaliana* gene identifiers showing changes in both protein accumulation (Lan et al., 2011) and gene expression (Rodríguez-Celma et al., 2013b)**.
