# Supplementary material for: Iron deficiency in plants: an insight from proteomic approaches
Source: Front Plant Sci. 2013 Jul 25;4:254. doi: 10.3389/fpls.2013.00254 (PMC3722493; doi:10.3389/fpls.2013.00254)
Supplement: Table S1 — Tables of BLAST results from proteins identified in roots from Prunus dulcis × Prunus persica (Rodríguez-Celma et al., 2013a), Medicago truncatula (Rodríguez-Celma et al., 2011a), Beta vulgaris (Rellán-Álvarez et al., 2010), Cucumis sativus (Donnini et al., 2010), and Solanum lycopersicum (Herbik et al., 1996; Brumbarova et al., 2008; Li et al., 2008). Spot nomenclature in the first column is that used in the original papers. BLAST searches were performed in the NCBI website (http://blast.ncbi.nlm.nih.gov/Blast.cgi) against the Arabidopsis database (taxid:3701) in March 2013. BLAST annotations were assigned as not hit when BLAST E-values were higher than 1e−30. If any of the accession numbers overlapped between two or more entries of the same plant species, they were considered redundant and only a single representative entry was retained. [file DataSheet1.pdf]

Table S1. Tables of BLAST results from proteins identified in roots from *Prunus dulcis x Prunus persica* (Rodríguez-Celma et al., 2013), *Medicago truncatula* (Rodríguez-Celma et al., 2011a), *Beta vulgaris* (Rellán-Álvarez et al., 2010), *Cucumis sativus* (Donnini et al., 2010), and *Solanum lycopersicum* (Brumbarova et al., 2008; Li et al., 2008; Herbik et al., 1996). Spot nomenclature in the first column is that used in the original papers. BLAST searches were performed in the NCBI website (http://blast.ncbi.nlm.nih.gov/Blast.cgi) against the Arabidopsis database (taxid:3701) in March 2013. BLAST annotations were assigned as not hit when BLAST E-values were higher than $1e^{-30}$. If any of the accession numbers overlapped between two or more entries of the same plant species, they were considered redundant and only a single representative entry was retained.

Table S2. Comparison of changes observed upon Fe deficiency in the non-redundant *Arabidopsis* root proteomes obtained from *Arabidopsis thaliana* (Lan et al., 2011) and from BLAST results in *Cucumis sativus*, *Solanum lycopersicum*, *Medicago truncatula* and *Prunus dulcis x Prunus persica*. Red and green backgrounds indicate increases and decreases, respectively, in protein abundance upon Fe deficiency. A blue background in the first column marks proteins showing changes in two or more plant species (not considering the *M. truncatula* and *B. vulgaris* treatments including $CaCO_3$, which are included in the Table in the last two columns in white characters).

Table S3. List of *Aradidopsis thaliana* gene identifiers showing changes in both protein accumulation (Lan et al., 2011) and gene expression (Rodriguez-Celma et al., 2013b).

Table S1. Tables of BLAST results from proteins identified in roots from *Prunus dulcis x Prunus persica* (Rodríguez-Celma et al., 2013), *Medicago truncatula* (Rodríguez-Celma et al., 2011a), *Beta vulgaris* (Rellán-Álvarez et al., 2010), *Cucumis sativus* (Donnini et al., 2010), and *Solanum lycopersicum* (Brumbarova et al., 2008; Li et al., 2008; Herbik et al., 1996). Spot nomenclature in the first column is that used in the original papers. BLAST searches were performed in the NCBI website (http://blast.ncbi.nlm.nih.gov/Blast.cgi) against the Arabidopsis database (taxid:3701) in March 2013. BLAST annotations were assigned as not hit when BLAST E-values were higher than $1e^{-30}$. If any of the accession numbers overlapped between two or more entries of the same plant species, they were considered redundant and only a single representative entry was retained.

Proteins showing changes in roots of a Prunus rootstock, *Prunus dulcis x Prunus persica*, **as a result of Fe-deficiency as described in Rodríguez-Celma et al., 2013. Forty spots in total; 17 different spots were found to change with Fe-deficiency, in bold in the left part of the second column, all of them identified as follows:**

Spot	-Fe vs. +Fe/-FeR vs. +Fe	Protein name	ID	Species	ATG	description	gene name	E value
**Oxidative stress**								
1	**new / new**	peroxidase	Q3S615	*Phaseolus lunatus*	At1g71695	peroxidase 12	PER12	1.0×10-127
4	**2.2** / **2.3**	peroxidase	Q3S615	*Phaseolus lunatus*	At1g71695	peroxidase 12	PER12	1.0×10-127
5	**2.0** / **2.1**	peroxidase	Q43854	*Vigna angularis*	At1g71695	peroxidase 12	PER12	1.0×10-148
8	1.6 / **2.0**	peroxidase	Q94IQ1p	*Prunus persica*	At1g71695	peroxidase 12	PER12	1.0×10-154
9	-2 / -2.5	peroxidase	Q94IQ1p	*Prunus persica*	At1g71695	peroxidase 12	PER12	1.0×10-154
2	**2.8** / 1.7	manganese superoxide dismutase	Q9G2T0	*Prunus persica*	At3g10920	Superoxide dismutase [Mn] 1, mitochondrial	MSD1	3.0×10-53
3	**2.5** / **2.6**	L-galactose dehydrogenase	B6ZL95	*Prunus persica*	At4g33670	L-galactose dehydrogenase	LGALDH	0
6	1.8 / **2.2**	copper/zinc superoxide dismutase	A8UDS9	*Nicotiana tabacum*	At1g08830	Superoxide dismutase [Cu-Zn] 1	CSD1	1.0×10-50
7	1.7 **/ 2.0**	thioredoxin H	Q93WZ3	*Prunus persica*	At3g51030	Thioredoxin H1	TRX1	7.0×10-55
10	-1.7 / -2.5	catalase	Q7XTK8	*Prunus persica*	At4g35090	Catalase-2	CAT2	0
**Plant stress and defense**								
11	**2.0** / **2.4**	(+)-neomenthol dehydrogenase-like	D7UC32p	*Prunus persica*	At3g61220	(+)-neomenthol dehydrogenase	SDR1	1.0×10-138

12	2.0 / **2.2**	glutathione-s-transferase omega, putative	B9SGT4p	*Prunus persica*	At5g02790	Glutathione S-transferase L3	GSTL3	1.0×10-88
13	1.5 / **2.3**	adenine nucleotide alpha hydrolases-like	G5DW21p	*Prunus armeniaca*	At1g11360	Adenine nucleotide alpha hydrolases-like protein		2.0×10-96
14	**-3.3 / -5**	major allergen Pru av 1	O24248	*Prunus persica*	At1g24020	MLP-like protein 423	MLP423	7.00E-12
15	-3.3 / -1.7	putative allergen Pru du 1.04	B6CQS3	*Prunus dulcis x Prunus persica*	At5g45860	abscisic acid receptor PYL11	PYL11	2.00E-05
17	**-2.5 / -1.7**	putative allergen Pru du 1.04	B6CQS3	*Prunus dulcis x Prunus persica*	At5g45860	abscisic acid receptor PYL11	PYL11	2.00E-05
16	**-3.3 / -2.5**	glutathione S-transferase	Q06FE1p	*Prunus persica*	At2g30860	Glutathione S-transferase F9	GSTF9	1.0×10-109
18	-1.3 / -2.5	glutathione S-transferase F1	Q6XX18p	*Prunus cerasus*	At1g02950	Isoform 2 of Glutathione S-transferase F4	GSTF4	1.0×10-55
**Protein synthesis/modification**								
19	**2.4** / 2.7	putative luminal-binding protein	Q0ZUG6	*Isatis tinctoria*	At5g42020	Mediator of RNA polymerase II transcription subunit 37f (Hsp70-12)	HSP70-11	0
20	1.7 / **2.0**	translation initiation factor 5A	Q9M5P9	*Euphorbia esula*	At1g13950	Eukaryotic translation initiation factor 5A-1	F16A14.17	6.0×10-94
21	1.0 / **2.4**	40S ribosomal protein S12	D3Y1X9p	*Prunus persica*	At2g32060	40S ribosomal protein S12-2	RPS12C	6.0×10-40
22	-1.4 / 2.2	alpha chain of nascent polypeptide associated complex	Q9M612p	*Prunus persica*	At3g12390	Nascent polypeptide-associated complex subunit alpha-like protein 1	T2E22.29	2.0×10-93
23	-1.1 / -2	calreticulin-3	G7KRL3p	*Prunus persica*	At1g08450	Calreticulin-3	CRT3	0

**Nitrogen metabolism**								
24	1.4 / **-5.0**	putative plastidic glutamine synthetase	Q8GUZ6	*Crataegus crus-galli*	At5g35630	Glutamine synthetase, chloroplastic/mitochondrial	GLN2	0
25	-2 / -2	nitrite reductase	Q93XS0	*Prunus persica*	At2g15620	Ferredoxin--nitrite reductase, chloroplastic	NIR1	0
26	-1.4 / -2.5	ferredoxin-nitrite reductase, putative	B9RYH9	*Ricinus communis*	At2g15620	Ferredoxin--nitrite reductase, chloroplastic	NIR1	0
27	-1.3 / -2	isoflavone reductase-like protein 6	Q3KN67	*Vitis vinifera*	At4g39230	NAD(P)H oxidoreductase, isoflavone reductase-like protein	T22F8.130	1.0×10-168
**Carbon metabolism**								
28	**2.3** / 1.5	enolase	Q1X8N5	*Prunus armeniaca*	At2g36530	Enolase	ENO2	2.0×10-89
29	**2.2** / 1.8	alcohol dehydrogenase	F6K5V5	*Prunus dulcis x Prunus persica*	At1g77120	Alcohol dehydrogenase class-P	ADH1	0
30	1.2 / **-2.0**	dihydrolipoamide acetyltransferase	E5GB89	*Cucumis melo subsp. melo*	At1g54220	Pyruvate dehydrogenase complex component E2 3	F20D21.4	0
31	-1.3 / -2.5	enolase	E6NU46	*Jatropha curcas*	At1g74030	Enolase 1, chloroplastic	ENO1	0
32	-1.1 / 2	ribose 5-phosphate isomerase A	Q9S726p	*Prunus persica*	At3g04790	Putative ribose 5-phosphate isomerase	F7O18.28	0
**Energy metabolism**								
33	**2.3** / **2.9**	flavodoxin-like quinone reductase 1	D7MUA0	*Arabidopsis lyrata subsp. lyrata*	At5g54500	1,4-benzoquinone reductase-like protein	FQR1	1.0×10-143

34	-1.4 / -3.3	quinone reductase family protein	D7MEH6p	*Prunus persica*	At4g27270	Quinone reductase family protein	AT4G27270	1.0×10-144
**Secondary metabolism**								
35	**3.0** / 1.8	phenazine biosynthesis protein, putative	B9S448p	*Prunus persica*	At4g02860	Similar to PHZF, catalyzing the hydroxylation of phenazine-1-carboxylic acid to 2-hydroxy-phenazine-1-carboxylic acid	T5J8.18	1.0×10-122
36	2.3 / **2.5**	isopentenyl-diphosphate Delta-isomerase I	O48964	*Camptotheca acuminata*	At5g16440	Isopentenyl-diphosphate Delta-isomerase I, chloroplastic	IPP1	1.0×10-148
37	1.2 / -1.7	chalcone synthase	Q76K34	*Prunus persica*	AT5G13930	chalcone synthase	TT4	5.0x10-82
**Others**								
38	**-5 / -1.4**	plastid-dividing ring protein similar to HSP70	Q6J4T5	*Solanum tuberosum*	At5g55280	Cell division protein FtsZ homolog 1, chloroplastic	FTSZ1	0
39	**-2.5 / -2.5**	BU042094 (poly(A)-binding protein, partial)	Q9M6E4p	*Prunus persica*	At1g49760	Poly(A) binding protein 8	PAB8	0
40	**-2 / -3.3**	B-glucosidase, putative	B9RIY8	*Ricinus communis*	At5g64570	B-D-xylosidase 4	BXL4	0

Proteins showing changes in roots of *Medicago truncatula* as a result of Fe-deficiency as described in Rodríguez-Celma et al., 2011. Fifty-six spots in total; 31 different spots were found to change with Fe-deficiency, from which 21 were identified as follows:

Spot	-Fe/-FeC vs +Fe	Protein name	ID	Species	ATG	description	gene name	E value
**Protein metabolism**								
1	11 / 6	cysteine protease	Q9STA4	*Medicago sativa*	At4g39090	Cysteine proteinase RD19a	RD19A	1.0×10-121
2	+3 / -	cysteine protease	Q9STA4	*Medicago sativa*	At4g39090	Cysteine proteinase RD19a	RD19A	1.0×10-121
3	- / New	proteasome subunit	O48551	*Glycine max*	At5g35590	Proteasome	PAA1	1.0×10-158

		alpha type-6				subunit alpha type-6-A		
4	- / +2	TPR1-Heat shock chaperone	Q7Y0Z0	*Medicago sativa*	At4g12400	Stress-induced protein sti1-like protein	T4C9.240	0
5	-2 / -	proteasome subunit alpha type 7	O24616	*Medicago truncatula*	At5g66140	proteasome subunit alpha type-7-B	PAD2	2E-153
6	- / -5	peptidase A1, pepsin	Q2HRQ7	*Medicago truncatula*	At1g11910	Aspartic proteinase A1	APA1	0
7	- / -7	similar to hypothetical trypsin/protease inhibitor	GT137702	*Medicago truncatula*	no hit			
8	- / -7	proteasome subunit β type-1	O82531	*Petunia x hybrida*	At3g60820	Proteasome subunit β type-1	PBF1	1.0×10-140
9	- / Lost	similar to hypothetical trypsin/protease inhibitor	BQ155019	*Medicago truncatula*	no hit			
10	Lost / Lost	GroEL-like chaperone, ATPase	Q1RSH4	*Medicago truncatula*	At3g23990	Chaperonin CPN60, mitochondrial	CPN60	0
**N metabolism**								
11	New / -	Putative S-adenosylmethionine-dependent methyltransferases	Q9C9Q8	*Arabidopsis thaliana*	At1g78240	Probable pectin methyltransferase QUA2	QUA2	-
12	New / New	UREG (urease accessory protein G)	O64700	*Arabidopsis thaliana*	At2g34470	Putative urease accessory protein	UREG	-
13	2 / 2	Chain A, dihydrolipoamide Dehydrogenase Of Glycine Decarboxylase	Q9M5K3	*Pisum sativum*	At1g48030	dihydrolipoyl dehydrogenase 1	LPD1	0
14	- / +2	aspartate aminotransferase	Q40325	*Medicago sativa*	At4g31990	Aspartate aminotransferase, chloroplastic	ASP5	0
15	- / -2	serine	Q45FE6	*Medicago*	At5g26780	Serine	SHM2	0

		hydroxymethyltransferase		*truncatula*		hydroxymethyltransferase		
16	- / -3	glutamine synthetase	Q84UC1	*Medicago truncatula*	At5g35630	Glutamine synthetase, chloroplastic/mitochondrial	GLN2	0
17	- / -4	OAS-TL4 cysteine synthase	A5YT88	*Glycine max*	At3g59760	Cysteine synthase, mitochondrial	OASC	0
18	- / -5	nitrite reductase	Q41099	*Phaseolus vulgaris*	At2g15620	Ferredoxin--nitrite reductase, chloroplastic	NIR1	0
19	- / Lost	S-adenosyl-L-methionine synthetase	A4ULF8	*Medicago sativa*	At3g17390	S-adenosylmethionine synthase 4	METK4	0
**C metabolism**								
20	New / New	putative fructose bisphosphate aldolase	Q45NN5	*Medicago sativa*	At5g03690	Fructose-bisphosphate aldolase		1.0×10^{-53}
21	New / New	glyceraldehyde-3-phosphate dehydrogenase	A5A4G4	*Medicago sativa*	At1g13440	Glyceraldehyde-3-phosphate dehydrogenase 2, cytosolic	GAPC2	1.0×10^{-128}
22	3 / 4	putative fructose bisphosphate aldolase	Q45NN5	*Medicago sativa*	At2g36460	Fructose-bisphosphate aldolase		1.0×10^{-53}
23	+3 / -	glyceraldehyde-3-phosphate dehydrogenase	A5A4G4	*Medicago sativa*	At1g13440	Glyceraldehyde-3-phosphate dehydrogenase 2, cytosolic	GAPC2	1.0×10^{-128}
24	- / +2	phosphoglycerate mutase	Q7XYD2	*Triticum aestivum*	At3g08590	Probable 2,3-bisphosphoglycerate-independent phosphoglycerate mutase 2	F17O14.6	1.0×10^{-160}
25	- / +2	fructose-bisphosphate aldolase, cytoplasmic isozyme	O65735	*Cicer arietinum*	At5g03690	Fructose-bisphosphate aldolase	F17C15_110	0
26	- / +2	enolase	Q6RIB7	*Glycine max*	At2g36530	Bifunctional	ENO2	0

						enolase 2/transcriptional activator		
27	- / -2	fructokinase-like protein	Q8LPE5	*Cicer arietinum*	At1g06030	Probable fructokinase-2	T21E18.8	1.0×10^{-143}
28	- / New	succinyl-CoA ligase β subunit	A5HIG2	*Caragana jubata*	At2g20420	Succinyl-CoA synthetase β chain	F11A3.3	0
29	9 / 7	hydroxyacylglutathione hydrolase	Q45NN8	*Medicago sativa*	At3g10850	Hydroxyacylglutathione hydrolase cytoplasmic	GLX2-2	7.0×10^{-99}
30	7 / 6	cytosolic NADP-malic enzyme	A6XB67	*Malus x domestica*	At1g79750	NADP-dependent malic enzyme 4, chloroplastic	NADP-ME4	0
31	- / +3	2-oxoacid dehydrogenase family protein	Q9FLQ4	*Arabidopsis thaliana*	At5g55070	2-oxoglutarate dehydrogenase complex component E2-1	MCO15.2	-
**Energy**								
32	- / -2	EMB1467 (EMBRYO DEFECTIVE 1467); NADH dehydrogenase	Q9FGI6	*Arabidopsis thaliana*	At5g37510	NADH dehydrogenase [ubiquinone] iron-sulfur protein 1, mitochondrial	MPA22.5	-
33	- / -2	ATRFNR2 (ROOT FNR 2)	Q9S9P8	*Arabidopsis thaliana*	At1g30510	Ferredoxin--NADP reductase, root isozyme 2, chloroplastic	RFNR2	-
34	-3 / -5	ATRFNR2 (ROOT FNR 2)	Q9S9P8	*Arabidopsis thaliana*	At1g30510	Ferredoxin--NADP reductase, root isozyme 2, chloroplastic	RFNR2	-
35	-3 / -5	ATRFNR2 (ROOT FNR 2)	Q9S9P8	*Arabidopsis thaliana*	At1g30510	Ferredoxin--NADP reductase, root isozyme 2, chloroplastic	RFNR2	-
36	Lost / -12	76 kDa mitochondrial	Q43644	*Solanum*	At5g37510	NADH	EMB1467	0

		complex I subunit		*tuberosum*		dehydrogenase [ubiquinone] iron-sulfur protein 1, mitochondrial		
37	-6/ Lost	quinonprotein alcohol dehydrogenase-like	A2Q5S2	*Medicago truncatula*	no hit			
**Secondary metabolism**								
38	New / New	NAD-dependent epimerase/dehydratase	A2Q3W4	*Medicago truncatula*	At2g45400	Dihydroflavonol 4-reductase-like protein	BEN1	6.0×10^{-99}
39	New / New	DMRL_synthase	B7FHG0	*Medicago truncatula*	At2g44050	6,7-dimethyl-8-ribityllumazine synthase, chloroplastic	F6E13.18	5.0×10^{-72}
40	- / New	GTP cyclohydrolase II/3,4-dihydroxy-2-butanone 4-phosphate synthase	Q6A4W9	*Malus x domestica*	At5g64300	Riboflavin biosynthesis protein ribBA, chloroplastic	RIBBA	0
41	- / New	GTP cyclohydrolase II/3,4-dihydroxy-2-butanone 4-phosphate synthase	Q6A4W9	*Malus x domestica*	At5g64300	Riboflavin biosynthesis protein ribBA, chloroplastic	RIBBA	0
42	- / -4	chalcone-flavanone isomerase	B7FJK3	*Medicago truncatula*	At3g55120	Chalcone--flavonone isomerase 1	CHI1	5.0×10^{-93}
**Stress**								
43	7 / 6	glutathione transferase	O49821	*Carica papaya*	At1g78380	Glutathione S-transferase U19	GSTU19	1.0×10^{-118}
44	+3 / -	manganese superoxide dismutase-like protein	B2BDZ8	*Pistacia vera*	At3g10920	Superoxide dismutase [Mn] 1, mitochondrial	MSD1	1.0×10^{-133}
45	- / +4	monodehydroascorbate reductase, seedling isozyme	Q42711	*Cucumis sativus*	At3g52880	Monodehydroascorbate reductase (NADH)	MDAR1	0
46	- / +2	thaumatin-like protein PR-5b	O81926	*Cicer arietinum*	At4g11650	Osmotin-like protein OSM34	OSM34	1.0×10^{-127}

47	- / -2	peroxidase	Q40366	*Medicago sativa*	At5g06720	Peroxidase 53	PER53	1.0×10^{-123}
48	- / -2	superoxide dismutase [Cu-Zn], chloroplast precursor	O65198	*Medicago sativa*	At2g28190	Superoxide dismutase [Cu-Zn] 2, chloroplastic	CSD2	1.0×10^{-100}
49	- / -2	L-ascorbate peroxidase	Q45NL3	*Medicago sativa*	At3g09640	L-ascorbate peroxidase 2, cytosolic	APX2	6.0×10^{-57}
50	- / -3	Superoxide dismutase [Cu-Zn]	Q02610	*Pisum sativum*	At1g08830	Superoxide dismutase [Cu-Zn] 1	CSD1	1.0×10^{-88}
51	- / -12	similar to lipid-associated family protein	I1KT36	*Glycine max*	AT2G22170	PLAT-plant-stress domain-containing protein	AT2G22170	$1\text{x}10^{-64}$
52	- / Lost	cold shock protein		*Medicago truncatula*	no hit			
**Others**								
53	- / +3	Alpha-1,4-glucan-protein synthase [UDP-forming] 2	Q8RU27	*Ricinus communis*	At5g15650	UDP-arabinopyranose mutase 2	RGP2	0
54	- / -3	predicted protein	A9TPK9	*Physcomitrella patens*	At4g34110	Polyadenylate-binding protein 2	PAB2	0
55	- / -6	guanine nucleotide-binding protein subunit β-like protein	O24076	*Medicago sativa*	At3g18130	Guanine nucleotide-binding protein subunit β-like protein C	RACK1C	0
56	- / Lost	annexin	Q42922	*Medicago sativa*	At5g65020	Annexin D2	ANN2	1.0×10^{-156}

Proteins showing changes in roots of *Beta vulgaris* as a result of Fe-deficiency as described in Rellán-Álvarez et al., 2010. Sixty-one spots in total were found to change with Fe-deficiency, from which 22 were identified as follows:

Spot	Protein name	ID	Species	ATG	description	gene name	E value
			Increased proteins in Fe-deficiency				
a	fructose 1,6-bisphosphate aldolase	T48396	*A. Thaliana*	At5g03690	fructose 1,6-bisphosphate aldolase	FBA4	-

b	triose-phosphate isomerase	gi|556171	*C. japonica*	At3g55440	Triosephosphate isomerase, cytosolic	CTIMC	1.0×10-153
c	cytosolic 3-phosphoglycerate kinase	gi|28172909	*T. Aestivum*	At1g79550	Phosphoglycerate kinase	PGK	0
d	enolase	gi|1087071	*M. crystallinum*	At2g36530	2-phosphoglycerate dehydratase 2	ENO2	0
e	enolase	T12341	*L. sativa*	At2g36530	2-phosphoglycerate dehydratase 2	ENO2	0
f	malate dehydrogenase	CAB61618	*B. vulgaris*	At1g04410	Malate dehydrogenase, cytoplasmic 1	F19P19.13	0
g	malate dehydrogenase	CAB61618	*B. vulgaris*	At1g04410	Malate dehydrogenase, cytoplasmic 1	F19P19.13	0
h	malate dehydrogenase	gi|48375044	*N. tabacum*	At3g15020	Malate dehydrogenase 2, mitochondrial	mMDH2	1.0×10-125
i	F1 ATPase α subunit	O78692	*B. vulgaris*	AtMg01190	ATP synthase subunit alpha, mitochondrial	ATPA	0
j	F1 ATPase ß subunit	gi|4388533	*S. bicolor*	At5g08690	ATP synthase subunit β-2, mitochondrial	T2K12.9	0
k	fructokinase	gi|1052973	*B. vulgaris*	At1g06030	Probable fructokinase-2	T21E18.8	0
l	formate dehydrogenase	gi|38636526	*Q. robur*	At5g14780	Formate dehydrogenase, mitochondrial	FDH1	0
m	At1g79210/YUP8H12R_1	gi|21689609	*A. thaliana*	At1g79210	proteasome_alpha_type_2		-
n	glycine rich protein	gi|16301	A. thaliana	At2g21660	Glycine-rich RNA-binding	RBG7	-

					protein 7		
New spots in Fe-deficiency							
o	glyceraldehyde 3-phosphate DH	gi|19566	*M. quinquepeta*	At1g13440	Glyceraldehyde-3-phosphate dehydrogenase 2, cytosolic	GAPC2	0
p	DMRL synthase	Q9XH32	*S. oleracea*	At2g44050	6,7-dimethyl-8-ribityllumazine synthase, chloroplastic	F6E13.18	6.0×10-85
Decreased spots in Fe-deficiency							
q	nucleoside diphosphate kinase I	gi|3309053	*M. crystallinum*	At4g09320	Nucleoside diphosphate kinase 1	NDPK1	7.0×10-89
r	oxalate oxidase-like germin 171	gi|11496133	*B. vulgaris*	At5g20630	Germin-like protein subfamily 3 member 3	GER3	2.0×10-60
s	At4g27270	gi|34365651	*A. thaliana*	At4g27270	Quinone reductase family protein		
Missing spots in Fe-deficiency							
t	oxalate oxidase-like germin 171	gi|11496133	*B. vulgaris*	At5g20630	Germin-like protein subfamily 3 member 3	GER3	2.0×10-60
u	peroxidase	gi|2956703	*S. oleracea*	At4g11290	Peroxidase 39	PER39	1.0×10-21
v	caffeoyl CoA O-methyltransferase	gi|5101868	Z. Mays	At4g34050	Caffeoyl-CoA O-methyltransferase 1	CCOAOMT1	1.0×10-154

Proteins showing changes in roots of *Cucumis sativus* as a result of Fe-deficiency as described in Donnini et al., 2010. Fifty-seven spots in total were found to change with Fe-deficiency, from which 44 different spots were identified as follows:

Spot ID	Protein name	ID	Species	ATG	description	gene name	Eblast
**Increased proteins in Fe deficiency**							
**Glycolysis**							
813	2,3-	Q42908	*Mesembryanthem*	At3g08590	2,3-	F17O14.6	0

	bisphosphoglycerate-independent phosphoglycerate mutase		*um crystallinum*		bisphosphoglycerate-independent phosphoglycerate mutase		
832	2,3-bisphosphoglycerate-independent phosphoglycerate mutase	O24246	*Prunus dulcis*	At1g09780	2,3-bisphosphoglycerate-independent phosphoglycerate mutase	PGM1	0
869	2,3-bisphosphoglycerate-independent phosphoglycerate mutase	P35493	*Ricinus communis*	At3g08590	2,3-bisphosphoglycerate-independent phosphoglycerate mutase	F17O14.6	0
954	pyrophosphate--fructose 6-phosphate 1-phosphotransferase subunit β	Q41141	*Ricinus communis*	At1g12000	Phosphofructokinase	PFP-BETA1	0
1080	Enolase	P42896	*Ricinus communis*	At2g36530	Enolase	ENO2	0
1116	LOS2	AAS66001	*Capsella bursa-pastoris*	At2g36530	Enolase	ENO2	0
1514	phosphoglycerate kinase, cytosolic	Q42962	*Nicotiana tabacum*	At1g79550	phosphoglycerate kinase	PGK	0
1612	fructose-bisphosphate aldolase	CAB77243	*Persea americana*	At5g03690	aldolase	F17C15_110	0
1662	fructose-bisphosphate aldolase	CAB77243	*Persea americana*	At5g03690	aldolase	F17C15_110	0
**Carbohydrate-related metabolism**							
1519	putative alcohol dehydrogenases	ABC02081	*Cucumis melo*	At1g77120	Alcohol dehydrogenase class-P	ADH1	0
1593	putative alcohol dehydrogenases	ABC02081	*Cucumis melo*	At1g77120	Alcohol dehydrogenase class-P	ADH1	0
1739	malate dehydrogenase, cytoplasmic	Q08062	*Zea mays*	At1g04410	Malate dehydrogenase,	F19P19.13	0

					cytoplasmic 1		
2613	galactokinase	ACJ04703	*Cucumis melo*	At3g06580		GAL1	0
**Nitrogen-related metabolism**							
1195	alanine aminotransferase	AAR05449	*Capsicum annuum*	At1g72330	Alanine aminotransferase 2, mitochondrial	ALAAT2	0
1321	S-adenosylmethionine synthetase 1	A9P822	*Populus trichocarpa*	At3g17390	S-adenosylmethionine synthase 4	METK4	0
1341	S-adenosylmethionine synthase	AAT40304	*Medicago sativa*	At1g02500	S-adenosylmethionine synthase 1	SAM1	0
1760	carbon-nitrogen hydrolase family protein	NP_196765	*Arabidopsis thaliana*	At5g12040	Omega-amidase		0
2607	glutamine synthetase cytosolic isozyme 1	P51118	*Vitis vinifera*	At5g35630	glutamine synthetase cytosolic isozyme 1	GLN2	0
**Redox-related and other proteins**							
724	heat shock protein 70	CAB72130	*Cucumis sativus*	At5g02500	heat shock protein 70	HSP70-1	0
858	protein disulfide isomerase (PDI)-like protein 2	AAU04766	*Cucumis melo*	At1g60420	Probable nucleoredoxin 1	T13D8.29	0
1515	old yellow enzyme-like*e*	CAN60665	*Vitis vinifera*	At1g76690	12-oxophytodienoate reductase 2	OPR2	0
			**decreased proteins in Fe deficiency**				
**Metabolism of sucrose and complex structural carbohydrates**							
586	invertase 2	ACJ04702	*Cucumis melo*	At1g12240	vacuolar invertase	BFRUCT4	0
588	invertase 2	ACJ04702	*Cucumis melo*	At1g12240	vacuolar invertase	BFRUCT4	0
596	invertase 2	ACJ04702	*Cucumis melo*	At1g12240	vacuolar invertase	BFRUCT4	0
712	xylan 1,4-β-xylosidase	CAJ65921	*Populus alba* x *Populus tremula*	At5g64570	B-D-xylosidase 4	BXL4	0

1169	predicted UDP-glucose 6-dehydrogenase*d*	CAN62897	*Vitis vinifera*	At3g29360	Probable UDP-glucose 6-dehydrogenase 1	UGD1	0
**Structural proteins**							
1113	β-tubulin	ABS50668	*Eucalyptus grandis*	At5g12250	Tubulin β-6 chain	TUBB6	0
1176	tubulin alpha-3 chain	P22275	*Zea mays*	At4g14960	Tubulin alpha-6 chain	TUBA6	0
1217	alpha-tubulin	AAO73546	*Ceratopteris richardii*	At1g04820	Tubulin alpha-4 chain	TUBA4	0
1433	actin	AAP73449	*Gossypium hirsutum*	At5g09810	Actin-7	ACT7	0
1442	actin	AAP73449	*Gossypium hirsutum*	At5g09810	Actin-7	ACT7	0
1454	actin	AAP73449	*Gossypium hirsutum*	At5g09810	Actin-7	ACT7	0
1676	actin	AAP73449	*Gossypium hirsutum*	At5g09810	Actin-7	ACT7	0
1438	actin	AAG10041	*Setaria italica*	At5g09810	Actin-7	ACT7	0
1637	globulin-like protein	AAF64423	*Cucumis melo*	At1g07750	Cupin domain-containing protein	F24B9.13	7.0×10-41
**Stress response proteins**							
757	heat shock protein 70	CAB72130	*Cucumis sativus*	At3g12580	Probable mediator of RNA polymerase II transcription subunit 37c	HSC70-4	0
758	heat shock protein 70	CAB72129	*Cucumis sativus*	At3g12580	Probable mediator of RNA polymerase II transcription subunit 37c	HSC70-4	0
**Fe containing proteins**							
343	aconitate hydratase, cytoplasmic	P49608	*Cucurbita maxima*	At2g05710	Aconitate hydratase 2, mitochondrial	ACO2	0

350	aconitase-iron regulated protein 1	AAC26045	*Citrus limon*	At2g05710	Aconitate hydratase 2, mitochondrial	ACO2	0
1543	peroxidase	AAA33129	*Cucumis sativus*	At5g06720	Peroxidase 53	PER53	1.0×10-115
**Other proteins**							
871	protein disulfide isomerase (PDI)-like protein 2	AAU04766	*Cucumis melo*	At1g60420	Probable nucleoredoxin 1	T13D8.29	0
1106	ATP synthase subunit β, mitochondrial	P19023	*Zea mays*	At5g08690	ATP synthase subunit β-2, mitochondrial	T2K12.9	0
1340	S-adenosylmethionine synthetase 1	A9P822	*Populus trichocarpa*	At4g01850	S-adenosylmethionine synthase 2	SAM2	0
2186	wali7-like protein*d*	CAN71784	*Vitis vinifera*	At3g22850	Aluminum induced protein with YGL and LRDR motif		1.0×10-127

Proteins showing changes in roots of *Solanum Lycopersicum* as a result of Fe-deficiency as described in Brumbarova et al., 2008. Twenty-four spots in total were found to change with Fe-deficiency, from which 21 different spots were identified as follows:

protein id	ratio -Fe/+Fe	Protein name	TC/EST	uniprot	ATG	description	gene name	E blast
1204	up	IAA6		Q38824	At1g52830	Auxin-responsive protein IAA6	IAA6	1.0×10-134
3005	up	citrate lyase		CITC_LEUMC	no hit			
5410	up	2-oxoglutarate-dependent dioxygenase		BAD98961	At1g06620	1-aminocyclopropane-1-carboxylate oxidase homolog 1	F12K11.24	1.0×10-139
5605	up	DnaJ protein homologue	BG642987	Q04960	At3g44110	Chaperone protein dnaJ 3	ATJ3	0
6108	up	osmotin OSML13 precursor		P50701	At4g11650	Osmotin-like protein OSM34	OSM34	1.0×10-117
7113	up	osmotin-like OSML13 precursor		P50701	At4g11650	Osmotin-like protein OSM34	OSM34	1.0×10-117

7509	down	catalase 2		AAD41256	At4g35090	catalase-2	CAT2	0
7707	up	subtilisin-like protease	TC173386	CAB67120	At5g67360	Subtilisin-like protease	ARA12	0
7801	up	Similar to At1g26110	TC178309		At1g26110	required for mRNA decapping	DCP5	0
8003	up	β5 proteasome subunit	TC143384	CAC43326	At3g26340	Proteasome subunit β type-5-B	PBE2	0
8010	down	germin		7447345	no hit			
8108	up	germin	TC182144	AAC78470	At1g09560	Germin-like protein subfamily 2 member 1	GLP4	9.0×10-89
8204	up	peroxidase	TC136771		AT1G71695	peroxidase 12	F14O23.6	5x10-26
8205	up	peroxidase	TC174970		no hit			
8212	up	peroxidase	TC136771		AT1G71695	peroxidase 12	F14O23.6	5x10-26
8303	up	peroxidase	TC142656	AAA65637	At1g05260	Peroxidase 3	PER3	1.0×10-150
8309	up	fructose 1,6-bisphosphate aldolase, cytoplasmic	TC136283	CAA61947	At5g03690	Fructose-bisphosphate aldolase	F17C15_110	0
8407	up	fructose 1,6-bisphosphate aldolase, cytoplasmic	TC181073	P08440	At5g03690	Fructose-bisphosphate aldolase	F17C15_110	0
8409	up	26S proteasome AAA ATPase subunit RPT4a		BAC23035	At5g43010	26S protease regulatory subunit 10B homolog A	RPT4A	0
7115	up	not identified						
3010	up	not identified						
2201	up	not identified						
8503	up	phospho-2-dehydro-3-deoxyheptonate aldolase		CAA79856	At4g33510	Phospho-2-dehydro-3-deoxyheptonate aldolase 2, chloroplastic	DHS2	0
1607	down	DS2	TC187253	Q2QJT5	no hit			

Proteins showing changes in roots of *Solanum Lycopersicum* as a result of Fe-deficiency as described in Li et al., 2008. Forty-one spots in total were found to change with Fe-deficiency, from which 26 different spots were identified as follows:

protein id	ratio -Fe/+Fe	Protein name	EC	uniprot	ATG	description	gene name	E blast
a1536	-1000	glucan endo-1,3-β-D-glucosidase	E3.2.2.39	CAA52871	At3g57260	Glucan endo-1,3-β-glucosidase, acidic isoform		1.0×10-120
a'968	2.09	enolase	E4.2.1.11	Q42887	At2g36530	Bifunctional enolase 2/transcriptional activator	ENO2	0
a'795	1.41	pepc	E4.1.1.32	P29196	At1g53310	Phosphoenolpyruvate carboxylase 1	PPC1	0
a673	-1.82	ribulosebiphosphate carboxylase	E4.1.1.39		ATCG00490		rbcL	
a1178	1.35	Putative citochrome P450	E.1.14.-	Q9M4X2	At3g14630	Cytochrome P450, family 72, subfamily A, polypeptide 9	CYP72A9	1.0×10-161
a675	1.32	ascorbate free radical reductase	E.1.14.-	Q43497	At3g52880	Monodehydroascorbate reductase (NADH)	MDAR1	0
a1575	-1000	acyl-glycerol-3-phosphate acyltransferase	E2.3.1.15	Q6UUW0	At1g32200	Glycerol-3-phosphate acyltransferase, chloroplastic	ATS1	1.0×10-159
a485	-1.92	3-deoxy-d-arabinoheptulosonate 7-phosphate synthase precursor	E2.5.1.54	P21357	At4g33510	Phospho-2-dehydro-3-deoxyheptonate aldolase 2, chloroplastic	DHS2	0
a824	-1.73	acetolactase synthase, small subunit putative	E2.2.1.6S	Q9SMC2	At2g31810	Acetolactate synthase small subunit 2	F20M17.15	0
a370	-1.82	catechol oxidase precursor	EC1.10.3.1	Q06355	no hit			
a'35	1.45	methionine synthase	E2.1.1.14	O50008	At5g17920	5-	CIMS	0

						methyltetrahydropteroyltriglutamate--homocysteine methyltransferase		
a993	1.35	vacuolar-type H+-ATPase subunit B3	E6.5.1.-	Q8W4E2	At1g20260	V-type proton ATPase subunit B3	VHA-B3	0
a171	1.37	Hsp70	E3.1.345	CAB72130	At5g02500		HSP70-1	
a836	-2.49	peptidylprolyl isomerase (cyclophilin)	E5.2.1.2	B6VB54	At5g13120	Peptidyl-prolyl cis-trans isomerase CYP20-2, chloroplastic	CYP20-2	1.0×10-117
a'350	1000	signal recognition particle, subunit SRP54	E3.4.23.36	P49972	At1g48900	Signal recognition particle 54 kDa protein 3		0
a1299	-1000	ribonuclease II-like protein	E3.1.13.1	Q6NQJ6	At5g02250	Ribonuclease II, chloroplastic/mitochondrial	SRP-54C	0
a769	3.12	UDP-glucose:protein transglucosylase-like	E1.14.18.2	Q6IV07	At3g02230	UDP-arabinopyranose mutase 1	RGP1	0
a984	1.54	xyloglucan endotransglucosylase/hydrolase 2	E1.14.18.2	Q9SV60	At4g13090	Xyloglucan endotransglucosylase/hydrolase protein 2	XTH2	0
a633	1.32	mitogen-activated protein kinase 1		Q39021	At1g10210	Mitogen-activated protein kinase 1	MPK1	0
a'270	1.4	MAP kinase 4		Q39024	At4g01370	Mitogen-activated protein kinase 4	MPK4	0
a'637	1.97	signal transducer			unknown			
a956	1.63	oxidoreductase			unknown			
a110	1.34	phospholipase PLDa1		Q9AWC0	At1g52570	Phospholipase D alpha 2	PLDALPHA2	0
a1494	-100	DEAD box RNA helicase			unknown			
a893	1.5	annexin p34		O81536	At1g35720	Annexin D1	EAZ27071	1.0×10-148
a533	-100	hypothetical protein OSJ_010554			At3g57150	Putative uncharacterized protein		0

Proteins showing changes in roots of *Solanum Lycopersicum* as a result of Fe-deficiency as described in Herbik et al., 1996

protein id	ratio -Fe/+Fe	Protein name	uniprot	ATG	description	gene name	E blast
Herbik1	increase	GAPDH zea mays	P08735	At3g04120	Glyceraldehyde-3-phosphate dehydrogenase 1	GAPC1	0
Herbik2	increase	FDH solanum tuberosum	Q07511	At5g14780	Formate dehydrogenase, mitochondrial	FDH1	0
Herbik3	increase	AP Arabidopsis thaliana	F4HU93	At1g07890	L-ascorbate peroxidase	APX1	0

Table S2. Comparison of changes observed upon Fe deficiency in the non-redundant *Arabidopsis* root proteomes obtained from *Arabidopsis thaliana* (Lan et al., 2011) and from BLAST results in *Prunus dulcis x Prunus persica*, *Solanum lycopersicum*, *Medicago truncatula*, and *Cucumis sativus*. Red and green backgrounds indicate increases and decreases, respectively, in protein abundance upon Fe deficiency. A blue background in the first column marks proteins showing changes in two or more plant species (not considering the *B. vulgaris* and *M. truncatula* treatments including $CaCO_3$, which are included in the Table in the last two columns in white characters).

ATG	description	*Prunus*	*S. lycopersicum*	*M. truncatula* -Fe	*C. sativus*	*A. thaliana*	*M. truncatula* -Fe +$CaCO_3$	*B. vulgaris* -Fe +$CaCO_3$
**Carbon metabolism**								
At5g13110	glucose 6 phosphate dehydrogenase 2 G6PD2					At5g13110.1		
At1g24280	glucose 6 phosphate dehydrogenase 3 G6PD3					At1g24280.1		
At3g06580	galactokinase (GAL1)				ACJ04703			
At5g03690	fructose 1,6-bisphosphate aldolase (FBA4)		P08440 (8309/8407)		CAB77243	At5g03690.2	O65735	T48396
At2g36460	fructose-bisphosphate aldolase			Q45NN5			Q45NN5	
At3g55440	triosephosphate isomerase, cytosolic (CTIMC)							AAB62730
At2g36530	enolase (ENO2)	Q1X8N5	Q42887 (a'968)		P42896		Q6RIB7	Q43130
At1g79550	PGK				Q42962			AAO32640
At1g06030	fructokinase-2 putative						Q8LPE5	Q42645
At1g13440	glyceraldehyde-3-phosphate dehydrogenase 2, cytosolic (GAPC2)			A5A4G4			A5A4G4	P26518
At3g04120	glyceraldehyde-3-phosphate dehydrogenase 1 (GAPC1)		P08735 (Herbik1)					
At3g08590	2,3-bisphosphoglycerate-independent phosphoglycerate mutase 2				Q42908		Q7XYD2	

At1g09780	2,3-bisphosphoglycerate-independent phosphoglycerate mutase (PGM1)				O24246			
At1g12000	pyrophosphate--fructose 6-phosphate 1-phosphotransferase subunit β (PFP-BETA1)				Q41141			
At1g54220	pyruvate dehydrogenase complex component E2 3	E5GB89						
At1g04410	MDH				Q08062			CAB61618
At3g15020	mMDH2							AAT42189
At2g20420	succinyl-CoA synthetase β chain						A5HIG2	
At2g05710	aconitate hydratase 2, mitochondrial (ACO2)				P49608			
At3g10850	hydroxyacylglutathione hydrolase cytoplasmic (GLX2-2)			Q45NN8			Q45NN8	
At1g79750	NADP-dependent malic enzyme 4, chloroplastic (NADP-ME)			A6XB67			A6XB67	
At5g55070	2-oxoglutarate dehydrogenase complex component E2-1						Q9FLQ4	
At5g14780	formate dehydrogenase, mitochondrial (FDH)		Q07511 (Herbik2)					Q7XHJ0
At1g77120	alcohol dehydrogenase (ADH1)	F6K5V5			ABC02081			
At1g53310	phosphoenolpyruvate carboxylase 1 (PPC1)		P29196 (a'795)					
**Metabolism of sucrose and complex structural carbohydrates**								
At2g31810	Acetolactate synthase small subunit 2		Q9SMC2					
At5g64570	1,4-β-D-xylosidase 4 (BXL4)	B9RIY8			CAJ65921			
At1g12240	invertase 2 (BFRUCT4)				ACJ04702			
At3g29360	UDP-glucose 6-dehydrogenase 1 (UGD1)				CAN62897			
At3g57260	glucan endo-1,3-β-glucosidase,		CAA52871					

	acidic isoform						
At1g07260	UDP-glucosyl transferaseE71C3					At1g07260.1	
At4g02290	glycosyl hydrolase 9B13					At4g02290.1	
At3g09220	laccase7					At3g09220.1	
At5g15650	UDP-arabinopyranose mutase 2 (RGP2)						Q8RU27
At3g02230	UDP-arabinopyranose mutase 1 (RGP1)		Q6IV07				
At4g13090	xyloglucan endotransglucosylase (XTH2)		Q9SV60				
At5g06860	PGIP1 (polygalacturonase-inhibiting protein1)					At5g06860.1	
At5g36890	β glucosidase 42					At5g36890.1	
At3g50740	UDP-glucosyl transferase 72E1					At3g50740.1	
At5g48930	hydroxycinnamoyl-CoA shikimate/ quinate hydroxycinnamoyl transferase					At5g48930.1	
At1g71100	RSW10					At1g71100.1	
**Oxidative stress**							
At1g60420	Probable nucleoredoxin 1				AAU04766		
At1g71695	PER12	Q3S615	8204/8212				
At1g05260	PER3		AAA65637				
At3g51030	TRX1	Q93WZ3					
At1g45145	LIV1					At1g45145.1	
At4g33670	L-galactose dehydrogenase (LGALDH)		B6ZL95				
At3g52880	MDAR1		a675			At3g52880.1	Q42711
At1g07890	APX1		F4HU93				

At3g10920	MnSOD-mit	Q9G2T0		B2BDZ8				
At1g08830	CuZnSOD 1	A8UDS9				At1g08830.1	Q02610	
At5g18100	CuZnSOD3					At5g18100.1		
At2g28190	CuZnSOD 2						O65198	
At3g09640	APX2						Q45NL3	
At4g35090	CAT-2	Q7XTK8	AAD41256					
At4g25100	FeSOD1					At4g25100.1		
At1g60420	Probable nucleoredoxin 1				AAU04766			
At3g01420	alpha-dioxigenase1, DIOX1					At3g01420.1		
At5g06720	PER53				AAA33129		Q40366	
At4g11290	PER39							O49941
At2g37130	PER21					At2g37130.1		
**Plant stress and defense**								
At4g12400	stress-induced protein						Q7Y0Z0	
At1g06620	2-oxoglutarate dioxygenase		BAD98961					
At3g61220	SDR1	D7UC32p						
At4g11650	OSM34		P50701				O81926	
At3g59930	defensin-like protein					At3g59930.1		
At1g54410	dehydrin family protein					At1g54410.1		
At1g09560	GLP5		AAC78470			At1g09560.1		
At5g02780	GSTL1					At5g02780.1		
At5g02790	GSTL3	B9SGT4						
At1g78380	GSTU19			O49821			O49821	
At1g17180	GSTU25							

						At1g17180.1		
At2g30860	GSTF9	Q06FE1						
At1g02950	GSTF4	Q6XX18						
At5g20630	GER3							Q9FPQ0
At1g18970	GLP4					At1g18970.1		
At1g24020	MLP423	O24248						
At2g22170	plant stress protein						I1KT36	
At4g33720	pathogenesis-related protein					At4g33720.1		
At5g52310	cold regulated 78-COR78					At5g52310.1		
At4g18360	(*S*)-2-Hydroxy-acid oxidase,					At4g18360.1		
At1g09020	SNF4					At1g09020.1		
**Protein metabolism/regulation**								
At1g69410	ELF5A-3					At1g69410.1		
At1g13950	ELF5A-1	Q9M5P9				At1g13950.1		
At2g05830	EFLF-2B					At2g05830.1		
At3g60240	EIF4G					At3g60240.2		
At3g05560	RPL22B					At3g05560.1		
At5g27770	RPL22C					At5g27770.1		
At5g08180	RPL7Ae					At5g08180.1		
At4g26300	arg-tRNA ligase					At4g26300.1		
At5g67360	subtilisin-like protease		CAB67120					

At4g39090	cysteine proteinase RD19a			Q9STA4			Q9STA4	
At5g12140	cystatin-1 (CYS1)					At5g12140.1		
At5g35590	proteasome subunit alpha type-6-A (PAA1)						O48551	
At1g79210	proteasome_alpha_type_2							gi|21689609
At3g26340	proteasome subunit β type-5-B (PBE2)		CAC43326					
At5g43010	26S protease regulatory subunit 10B homolog A (RPT4A)		BAC23035					
At5g66140	proteasome subunit alpha type-7-B (PAD2)			O24616				
At3g60820	proteasome subunit β type-1 (PBF1)						O82531	
At1g11910	aspartic proteinase A1 (APA1)						Q2HRQ7	
At3g23990	CPN60			Q1RSH4			Q1RSH4	
At3g12580	HSC70-4				CAB72129			
At1g08450	CRT3	G7KRL3						
At3g44110	chaperone protein dnaJ 3 (ATJ3)		Q04960					
At5g42020	HSP70-11	Q0ZUG6						
At5g02500	HSP70-1		CAB72130		CAB72130			
At1g10210	mitogen-activated protein kinase 1 (MPK1)		Q39021					
At4g01370	mitogen-activated protein kinase 4 (MPK4)		Q39024					
At4g05200	Cys rich- RLK 25					At4g05200.1		
At1g64370	unknown					At1g64370.1		
At3g27210	unknown					At3g27210.1		

At5g62740	HIR1					At5g62740.1	
At3g01290	band 7 family protein					At3g01290.1	
**Nitrogen metabolism**							
At5g35630	GLN2	Q8GUZ6			P51118		Q84UC1
At5g37600	GLN1					At5g37600.1	
At2g34470	putative urease accessory protein (UREG)			O64700			O64700
At1g48030	LPD1 (dihydrolipoyl dehydrogenase 1)			Q9M5K3			Q9M5K3
At5g12040	omega-amidase				NP_196765		
At4g31990	aspartate aminotransferase (ASP5)						Q40325
At1g72330	alanine aminotransferase 2, mitochondrial (ALAAT2)				AAR05449		
At1g78240	putative SAM-dependent methyltransferases			Q9C9Q8			
At4g14710	acireductone dioxygenase, ARD2					At4g14710.1	
At5g53850	haloacid dehalogenase-like hydrolase family protein					At5g53850.2	
At2g36880	METHIONINE ADENOSYLTRANSFERASE3, MAT3					At2g36880.1	
At3g17390	SAM synthase 4 (METK4)				A9P822		A4ULF8
At5g17920	5-methyltetrahydropteroyltriglutamate-homocysteine methyltransferase		O50008 (a'35)				
At1g02500	SAM1				AAT40304	At1g02500.1	
At4g01850	SAM2				A9P822	At4g01850.1	

At5g26780	serine hydroxymethyltransferase (SHM2)						Q45FE6	
At3g59760	cysteine synthase (OASC)						A5YT88	
At4g33510	phospho-2-dehydro-3-deoxyheptonate aldolase 2, chloroplastic (DHS2)		P21357 (a485)					
**Energy metabolism**								
At5g04750	F1F0-ATPase inhibitor protein, putative					At5g04750.1		
At2g27730	Unknown protein-photorespiration					At2g27730.1		
At2g47690	NADH-ubiquinone oxidoreductase-related					At2g47690.1		
At4g21105	cytochrome *c* oxidase/electron carrier					At4g21105.1		
At3g10860	ubiquinol-cytochrome *c* reductase complex ubiquinone-binding protein, putative					At3g10860.1		
At4g20150	mitochondrial respiratory chain					At4g20150.1		
At3g12900	2OG-Fe(II) oxygenase family protein[1,2,3,4,5]					At3g12900.1		
At5g54500	1,4-benzoquinone reductase-like protein (FQR1)	D7MUA0						
At4g27270	quinone reductase family protein	D7MEH6						Q6NQE2
At5g13120	peptidyl-prolyl cis-trans isomerase CYP20-2, chloroplastic		B6VB54 (a836)					
At2g15620	FNR chloroplastic (NIR1)	Q93XS0					Q41099	
At4g39230	NAD(P)H oxidoreductase, isoflavone reductase-like protein	Q3KN67						
At5g37510	NADH dehydrogenase [ubiquinone] iron-sulfur protein 1, mitochondrial			Q43644			Q43644	

At1g30510	ferredoxin--NADP reductase, RFNR2)			Q9S9P8			Q9S9P8	
AtMg01190	ATPA							O78692
At5g08690	ATPB				P19023			O24345
At1g20260	V-type proton ATPase subunit B3		Q8W4E2 (a993)					
At1g15690	ATAVP3					At1g15690.1		
At2g21410	VHA-A2					At2g21410.1		
**Secondary metabolism**								
At2g44050	6,7-dimethyl-8-ribityllumazine synthase, chloroplastic			B7FHG0			B7FHG0	Q9XH32
At2g45400	dihydroflavonol 4-reductase-like protein			A2Q3W4			A2Q3W4	
At5g64300	riboflavin biosynthesis protein ribBA, chloroplastic (RIBA)			Q6A4W9			Q6A4W9	
At3g55120	chalcone-flavonone isomerase 1						B7FJK3	
At4g34050	caffeoyl-CoA O-methyltransferase 1					At4g34050.1		Q9XGD6
At3g53260	phenylalanine ammonia-lyase 2, PAL2					At3g53260.1		
At1g51680	4-Coumarate-CoA ligase 1, 4CL1					At1g51680.1		
At3g21240	coumarate:COA ligase 2					At3g21240.1		
At3g13610	F6′H1					At3g13610.1		
At5g16440	isopentenyl-diphosphate Delta-isomerase I, chloroplastic	O48964						
At5g13930	chalcone synthase	Q76K34						
At4g02860	similar to PHZF, catalyzing the hydroxylation of phenazine-1-carboxylic acid to 2-hydroxy-	B9S448						

	phenazine-1-carboxylic acid						
At1g11680	CYP51					At1g11680.1	
At1g50510	indigoidine synthase A family protein					At1g50510.1	
**Structural proteins**							
At1g35720	annexin D1		O81536				
At3g46000	actin-depolymerizing factor 2					At3g46000.1	
At1g01750	actin-depolymerizing factor 11					At1g01750.1	
At5g12250	TUBB6				ABS50668		
At4g14960	TUBA6				P22275		
At1g04820	TUBA4				AAO73546		
At5g09810	ACT7				AAP73449		
At1g07750	cupin domain-containing protein				AAF64423		
At5g65020	annexin D2						Q42922
**Lipid metabolism**							
At1g54630	acyl carrier protein 3, ACP3					At1g54630.1	
At2g47140	short-chain dehydrogenase/reductase (SDR) family protein					At2g47140.1	
At1g52570	phospholipase D alpha 2		Q9AWC0				
At1g32200	glycerol-3-phosphate acyltransferase, chloroplastic (ATS1)		Q6UUW0				
At3g02620	acyl-desaturase					At3g02620.1	
**Transport**							

At2g18370	pathogenesis-related protein				At2g18370.1		
At5g58060	YKT61				At5g58060.1		
At3g07100	SEC24A				At3g07100.1		
At1g12110	nitrate transporter 1, ATNRT1				At1g12110.1		
At4g34740	CIA1				At4g34740.1		
At5g62600	transportin-SR-related				At5g62600.1		
At2g46520	cellular apoptosis susceptibility protein				At2g46520.1		
**Fe homeostasis**							
At4g31940	CYP82C4				At4g31940.1		
At1g01580	FRO2				At1g01580.1		
At1g56430	NAS4				At1g56430.1		
At5g01600	FER1				At5g01600.1		
At3g56090	FER3				At3g56090.1		
At4g04770	NAP1				At4g04770.1		
At1g32500	NAP6				At1g32500.1		
**Others**							
At3g14630	CYP72A9	Q9M4X2					
At1g76690	12-oxophytodienoate reductase 2 (OPR2)			CAN60665			
At2g21660	glycine-rich RNA-binding protein 7 (RBG7)						gi|16301
At3g29075	glycine-rich protein				At3g29075.1		

At1g52830	auxin-responsive protein IAA6		Q38824 (1204)				
At1g26110	required for mRNA decapping		spot 7801				
At1g48900	signal recognition particle 54 kDa protein 3		P49972 (a'350)				
At3g11750	FOLB1				At3g11750.1		
At3g07720	kelch repeat-containing protein[1,2,3,4,5]				At3g07720.1		
At5g19860	unknown				At5g19860.1		
At2g28430	unknown				At2g28430.1		
At5g17440	LUC7 N-terminal domain-containing protein				At5g17440.1		
At1g79280	nuclear pore anchor				At1g79280.1		
At3g03340	unfertilized embrio SAC6				At3g03340.1		
At2g45430	AT-hook motif nuclear-localized protein 22				At2g45430.1		
At5g55280	cell division protein FtsZ homolog 1, chloroplastic	Q6J4T5					
At1g49760	poly(A) binding protein 8	Q9M6E4p					
At5g02250	ribonuclease II, chloroplastic/mitochondrial		Q6NQJ6				
At4g34110	polyadenylate-binding protein 2 (PAB2)					A9TPK9	
At4g09320	nucleoside diphosphate kinase 1 (NDK1)						O81372
At3g18130	guanine nucleotide-binding protein subunit β-like protein C					O24076	
At3g22850	aluminum induced protein with YGL and LRDR motif			CAN71784			
At4g35360	pantothenate kinase family protein				At4g35360.1		

At2g22125	unknown	At2g22125.1
At3g01680	unknown	At3g01680.1
At2g24020	unknown	At2g24020.1
At4g32870	unknown	At4g32870.1
At5g14790	unknown	At5g14790.1
At2g03820	nonsense-mediated mRNA decay NMD3 family protein	At2g03820.1
At1g09770	cell division cycle 5	At1g09770.1
At5g49760	leucine-rich repeat family protein	At5g49760.1

Table S3. List of *Aradidopsis thaliana* gene identifiers showing changes in both protein accumulation (Lan et al., 2011) and gene expression (Rodriguez-Celma et al., 2013).

At1g01580	At1g56430	At3g09220	At4g04770	At5g13110
At1g02500	At1g71100	At3g11750	At4g14710	At5g36890
At1g07260	At2g05830	At3g12900	At4g25100	At5g37600
At1g08830	At2g22125	At3g13610	At4g31940	At5g48930
At1g09560	At2g36880	At3g21240	At4g32870	At5g49760
At1g11680	At2g37130	At3g50740	At4g33720	At5g52310
At1g17180	At2g45430	At3g52880	At4g34050	At5g53850
At1g18970	At3g01420	At3g53260	At4g34740	
At1g45145	At3g01680	At3g56090	At5g01600	
At1g51680	At3g07720	At4g02290	At5g02780	
